# Drug release profile of a novel exenatide long-term drug delivery system (OKV-119) administered to cats

**DOI:** 10.1186/s12917-024-04051-6

**Published:** 2024-05-18

**Authors:** Michael Klotsman, Wayne H. Anderson, Chen Gilor

**Affiliations:** 1 Okava Pharmaceuticals, San Francisco, CA USA; 2 Pulmonary and Critical Care Medicine, University of North Carolina at Chapel Hill, Chapel Hill, NC USA; 3 Department of Small Animal Clinical Sciences, College of Veterinary Medicine, University of Florida, Gainesville, FL USA

**Keywords:** Exenatide, Feline diabetes, Feline obesity, Adherence, Drug delivery

## Abstract

Beneficial weight-loss properties of glucagon-like peptide-1 receptor agonists (GLP-1RA) in obese people, with corresponding improvements in cardiometabolic risk factors, are well established. OKV-119 is an investigational drug delivery system that is being developed for the long-term delivery of the GLP-1RA exenatide to feline patients. The purpose of this study was to evaluate the drug release characteristics of subcutaneous OKV-119 implants configured to release exenatide for 84 days. Following a 7-day acclimation period, five purpose-bred cats were implanted with OKV-119 protypes and observed for a 112-day study period. Food intake, weekly plasma exenatide concentrations and body weight were measured. Exenatide plasma concentrations were detected at the first measured timepoint (Day 7) and maintained above baseline for over 84 Days. Over the first 28 days, reduced caloric intake and a reduction in body weight were observed in four of five cats. In these cats, a body weight reduction of at least 5% was maintained throughout the 112-day study period. This study demonstrates that a single OKV-119 implant can deliver the GLP-1RA exenatide for a months long duration. Results suggest that exposure to exenatide plasma concentrations ranging from 1.5 ng/ml to 4 ng/ml are sufficient for inducing weight loss in cats.

**Supplementary Information:**

The online version contains supplementary material available at 10.1186/s12917-024-04051-6.

## Introduction

In people, obesity prevalence rates have nearly tripled since 1975; it is now the most prevalent chronic disease worldwide [1–3]. An alarming increase in obesity prevalence rates in companion animals, attributed in part to shared environment and lifestyle elements between people and pets, have been reported [4–6]. With prevalence estimates of up to 40% in the domestic cat population, the need to manage overweight or obese cats is now one of the most common challenges encountered in veterinary medicine [7–10].

Obesity, defined as an accumulation of excess body fat, is a chronic disease associated with decreased life expectancy in both people and companion animals [7, 11–14]. The pathophysiology of obesity is complex and multifactorial [15]. Over time, excessive adiposity in people leads to an underlying inflammatory state, appetite dysregulation, insulin resistance, hypertension, and dyslipidemia [16]. The complications of obesity, such as type 2 diabetes [17], cardiovascular disease [18], and increased risk of death [14], are well established in people. Although less well characterized, obesity predisposes cats to many of the same conditions as in people [7].

New approaches to controlling excess body fat are needed [19]. Historically, the treatment of feline obesity focused exclusively on lifestyle modifications. Restricted caloric intake remains the cornerstone of weight-loss intervention in cats [20, 21], but given the reported increase in prevalence rates it is clear that alternative long-term body weight management strategies are needed.

GLP-1 receptor agonists (GLP-1RAs) are a therapeutic drug class that target pathways of endogenous nutrient-stimulated hormones [22, 23]. GLP-1RAs have been shown to beneficially reduce body weight and improve the cardiometabolic risk profile in diabetic and non-diabetic obese people, with a low risk of hypoglycemia or other serious adverse events (AEs) [19, 24–28]. The mechanisms related to weight loss with GLP-1RAs are incompletely understood, but are partly attributed to GLP-1 inhibitory effects on gastric emptying, postprandial glucagon release, and stimulation of hypothalamic satiety centers [22, 29].

GLP-1RAs may hold therapeutic potential in feline patients [23, 30–32]. Studies conducted in healthy, purpose-bred cats have shown that short-term administration of GLP-1ARs are correlated with weight loss [33, 34]; however, the weight-loss properties of GLP-1RAs administered to cats over a longer duration are not well characterized. To build off of prior work in which purpose-bred healthy cats were implanted with OKV-119, an investigational drug delivery system that was designed to deliver up to 30 days of the GLP-1RA exenatide [33], the present study evaluated OKV-119 prototypes configured to provide months-long drug delivery. The primary purpose of this study was to characterize exenatide plasma concentrations over a 112 day study period in healthy cats implanted with a single OKV-119 implant. Secondary objectives were to evaluate caloric intake and body weight following exposure to exenatide.

## Materials and methods

### Study design

Five purpose-bred neutered male cats (Marshall BioResources, North Rose, NY, USA) were enrolled in an open-label study. At the time of enrollment, cats were 28 months old, weighed (median, range) 5.61 kg (5.14 –7.38 kg), and were considered healthy based on physical examination. The general health of each animal was assessed during the course of the 112 day study period.

Prior to test article administration, the cats were placed in individual pens and acclimated for seven days in the room where they were to be housed for the duration of the study. To allow for the measurement of daily caloric intake, cats were individually housed in pens from Day − 6 to Day 28. Cats were fed a standardized pelleted feline diet (Purina Cat Indoor formula), with 90 g of fresh feed provided once daily in the morning. Cats were offered canned wet food (Friskies Meaty Bits) to induce eating if caloric intake was too restricted. After Day 28, cats were removed from individual pens and were collectively provided 450 g of the dry pelleted food for the remainder of the study period. The amount of food provided and consumed was recorded throughout the study. Water was supplied ad libitum. Daily physical exams were performed, while body weights were measured weekly. Weekly plasma samples were taken during the course of the study for the measurent of exenatide concentrations.

This study was conducted under the standard operating procedures of the testing facility and was in compliance with current recommendations for the Guide for the Care and Use of Laboratory Animals. The study protocol was approved by the AAALAC accredited test facility (IACUC protocol 22OKA038).

### OKV-119 investigational drug-delivery system

The overall design and primary working principle of the OKV-119 systems used in the study have been previously described [33]. OKV-119 prototypes used in the present study were configured to release a peak of approximately 100 mcg exenatide per day for up to 84 Days.

The insertion and removal techniques of OKV-119 have been previously described [33]. Briefly, cats were sedated with 2 mg/Kg xylazine and 1 mg/Kg ketamine and a 4 × 4 cm area was clipped and aseptically prepared on the dorsal lumbar area [Supplemental Fig. 1]. Once prepared, the implant was subcutaneously inserted and removed through a 2–3 mm incision. The procedure was routinely completed within 30 to 60 s.

### Blood sampling and assay conditions

Blood samples were collected once prior to implantation (Day 0) and then weekly for the duration of the study. All samples were collected after an overnight fast. The blood collection procedures and validation of the LC-MS/MS assay conditions have been previously described [33].

### Statistical analysis

Clinical parameters, AEs, exenatide plasma concentrations, and laboratory parameters were summarized descriptively (e.g., n, mean, standard deviation [SD], median, minimum, maximum) using commercially available computer software^1^. Data are presented as median [range]. For observations between Day 0 and Day 28, the correlation between weekly percent change in body weight and caloric intake measurements was assessed by Pearson’s correlation coefficient. Cohen’s d was calculated to compare the effect size of change in body weights from Day 0 and Day 112 [35].

## Results

### Tolerability and safety

**Fig. 1:**
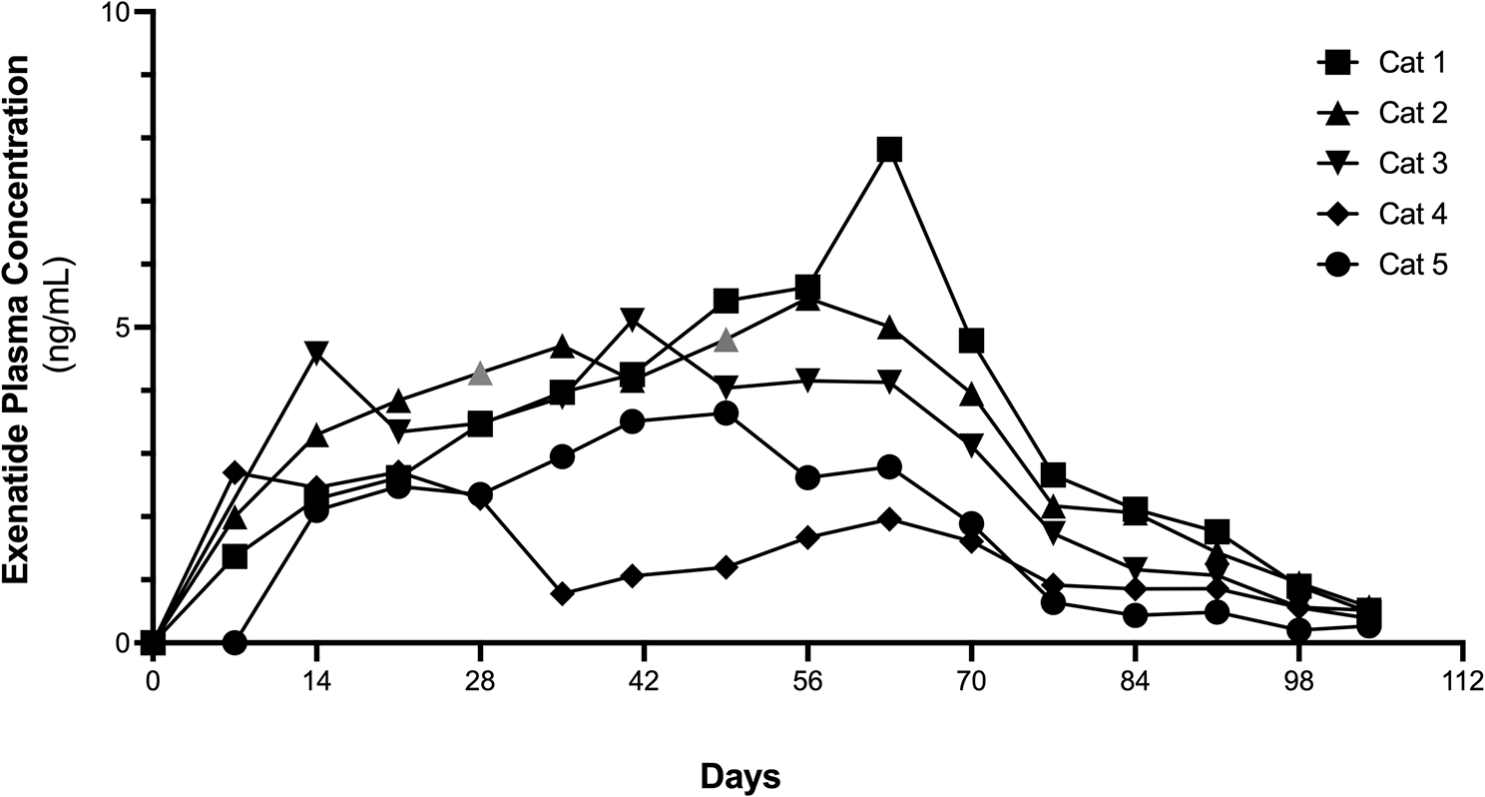
Exenatide plasma concentrations from baseline to Day 105 in purpose bred cats implanted with OKV-119 protypes configured to release exenatide for 84 Days

Cats appeared to tolerate the implants well. Signs of licking or scratching at the implant site were not observed, nor was there any visible evidence of inflammation in the skin overlying the implant (Fig. 1). From Days 1 to 7, Cat 1 experienced near complete anorexia and was offered canned wet food between Day 8 and Day 20 to facilitate eating. Based on daily health observations, there were no other apparent systemic drug-related adverse effects.

### Exenatide plasma drug concentrations

At baseline, plasma exenatide concentrations were below the level of detection in all cats. Exenatide plasma concentrations were detected at Day 7 and maintained above 1 ng/ml up to Day 70 (Fig. 1). Between Day 14 and Day 70, the median exenatide plasma concentration was 3.48 ng/ml (range: 0.78 ng/ml – 7.82 ng/ml). Exenatide plasma concentrations declined from Day 70 to Day 105 (Fig. 1).

### Caloric inkate and body weight

**Fig. 2:**
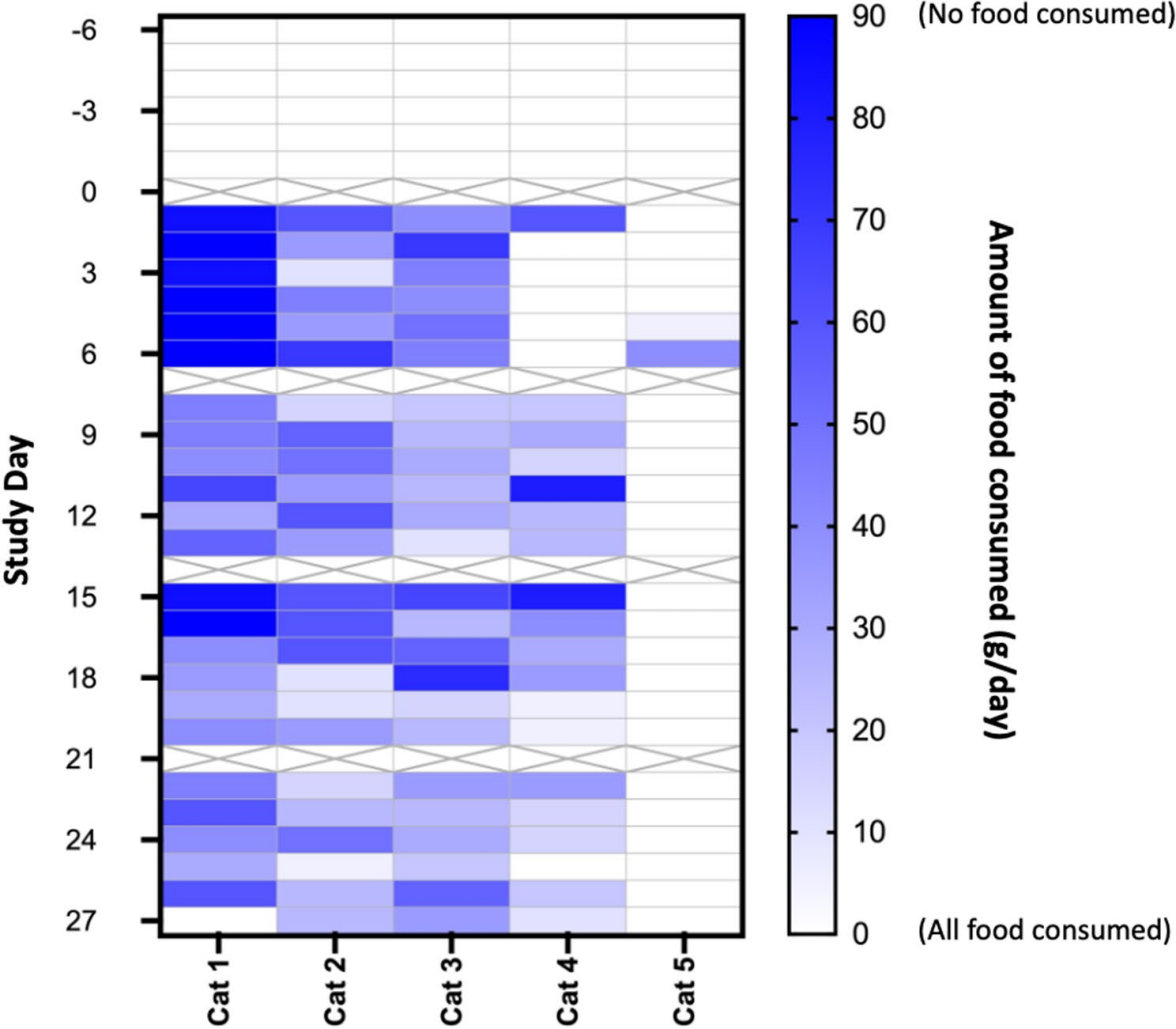
Heat map of food consumed by cats (*n* = 5) during the acclimation period (Days − 6 to Day − 1) and after insertion of the OKV-119 implant (Day 0 to Day 28). Each box represents the amount of food remaining (right y-axis) on a given day with study days (left y-axis). Columns correspond to each cat on the x-axis. Darker shades indicate less food consumed on a given day. Caloric intake was not reported on Days 0, 7, 14 and 28 (crossed-out boxes) because food was removed on the eve prior fasting blood draws

During the acclimation period (Day − 7 to Day 0), the cats consumed all of the daily food provided (Fig. 2). The median body weight for Cats 1–4 was 5.51 kg (range: 5.13 –5.97 kg) and remained stable from Day − 7 to Day 0 (mean difference = 0.03 kg; range: 0.01 –0.08 kg) (Table 1). Compared to Cats 1–4, Cat 5 was 39% heavier at Day − 7 and, in contrast to the other cats in this study, had a -3.6% decline in body weight between Day − 7 and Day 0 (Table 1).

**Table 1:** Baseline characteristics of five purpose-bred cats prior to the insertion of the OKV-119 systems

Cat Number	Body Weight (Kg)			Percent Change in Weight	
	Day − 7	Day 0	Day 112	Day − 7 to Day 0	Day 0 to Day 112
1	5.13	5.14	4.51	0%	-12%
2	5.47	5.41	4.89	-1	-10%
3	5.58	5.61	5.01	1%	-11%
4	5.97	5.89	5.50	-1	-7%
5	7.65	7.38	7.82	-4%	6%

**Fig. 3:**
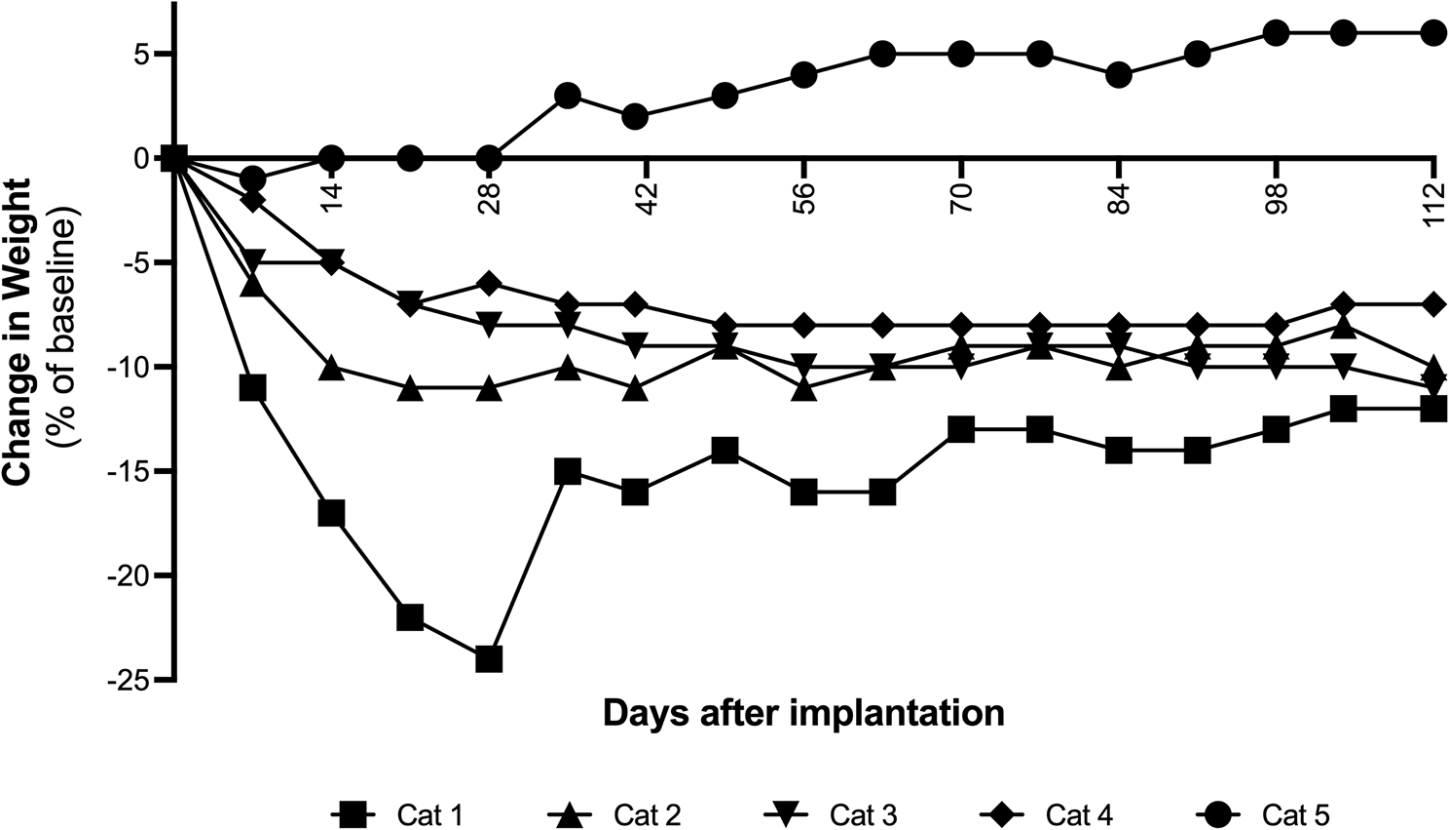
Percent change in body weight in five purpose bred cats from Day 0 to Day 112 following administration of a subcutaneous OKV-119 implant. Cats were housed in individual pens for the intitial 28 day study period, and then housed as a group for the remainder of the study period

From Day 0 to Day 28 reduced caloric intake was observed immediately following insertion of the OKV-119 implant in Cats 1–4 (Fig. 2). During this 28 day period, food intake was correlated to weekly declines in body weights (Pearson’s *r* = 0.49 [95% CI 0.12–0.74], *p* = 0.01). Body weights for Cats 1–4 were observed to decline for the first 28 days, afterwhich body weights remained stable to Day 112 (Fig. 3). In Cats 1–4, the mean decline in body weight from Day 0 to Day 112 was 0.54 kg (range: 0.39 –0.63 kg) (Cohen’s d = 4.9) (Fig. 3). The food consumption and body weight of Cat 5 was unchanged from Day 0 to Day 28. After transitioning out of an individual pen, Cat 5 was observed to gain 6.2% in body weight from Day 28 to Day 112 (Fig. 3).

## Discussion

To assess the feasibility the OKV-119 drug delivery system for use in cats, the initial study used implants configured to release exenatide for a 30-day period [33]. In the present study, we expand on that prior work and report the use of OKV-119 prototypes that released exenatide for over 84 days in purpose-bred cats. Immediately following exposure to exenatide, a reduction in caloric intake, with corresponding weight loss, was observed. The reduction in weight loss appeared to plateau by Day 28 and was then maintained for the duration of the 112 day study period. Weight loss with GLP-1RAs stems from a reduction in energy intake owing to a decreased appetite, which is thought to result from direct and indirect effects on the brain [19, 22].

Long-term positive energy balance that results in exssive accumulation of lipids is associated with a constellation of metabolic and pathphysologic abnormalities including impaired insulin signaling and insulin resistance, lipotoxicity, dyslipidemia (high plasma TG and low HDL-cholesteral concentrations), hypertension, and low-grade systemic inflammation [7, 13, 36]. Metabolic abnormalities in people with obesity have important clinical implications as the risk of developing cardiometabolic diseases is directly related to the number and severity of metabolically unhealthy phenotypes expressed [37].

In people, a moderate reduction (5–10%) in body weight is associated with clinical improvements in lipid profiles (e.g., triglicerides [TG], total cholesterol [TC]), other obesity-related cardiometabolic risk factors (e.g., insulin, fasting plasma glucose levels [FPG]), and descreases the risk of heart failure, stroke, chronic kidney disease, diabetes, and obstructive sleep apnea [38, 39]. Clinical trials in obese people with and without diabetes have shown that GLP-1RAs, the most recent anti-obesity medications approved for human use, are associated with beneficial weight-loss and metabolic improvements inclucing lower TC, TG, LDL, FPG, and insulin [27, 40–44]. Although GLP-1RAs have revolutionized the treatment of obesity in people, achieving long-term patient adherence is challenging because these drugs are typically administered as once-weekly injections [24–28]. Unlike other treatment modalities, the OKV-119 drug delivery system offers the dual benefit of guaranteed patient adherence vis-à-vis long-term drug delivery with a single implant and immediate cessation of drug release (i.e., removal of the implant) if adverse effects are observed [33].

Although less well documented, cats share many of the same pathophysiologic obesity phenotypes that afflict people [45]. It has been reported that compared to non-obese cats, obese cats have an altered metabolic profile characterized by higher insulin, glucose, TG, and inflammatory markers, and lower adiponectin levels [7, 13, 46–48]. However, the risk of developing cardiometabolic diseases relative to the number and severity of metabolically unhealthy phenotypes expressed in cats remains poorly defined. For example, although a body weight reduction of at least 5% is the threshold used by the FDA to define a clinically meaningful effect for both people and pets [49], at present there are no data regarding cardiometabolic health outcomes in cats with sustained weight loss. Larger prospective studies of longer duration in clinically obese cats will be required to establish whether, as reported in people, weight loss of any specific magnitude leads to metabolic improvements [27, 50, 51].

The data presented herein suggest that exenatide plasma concentrations ranging from 1.5 ng/ml to 4 ng/ml are sufficient for inducing weight loss in most cats. These observations are consistent with other studies which demonstrate the weight-loss effects of GLP-1RAs in cats [22, 31, 33, 34, 52]. Four of five cats in this study were observed to reduce their caloric intake and subsequently lose weight when exposed to exenatide; it remains unclear why the largest of the five cats was not observed to reduce their caloric intake or to lose weight. The target exenatide plasma concentrations needed for achieving weight loss in clinically obese household cats remains to be determined. Future studies are also needed to evauate whether beneficial weight losss associated with GLP-1ARs is also correlated with improvements in obesity-related cardiometabolic markers such as fasting insulin and FPG, TG, TC.

### Electronic supplementary material

Below is the link to the electronic supplementary material.

Supplementary Material 1

## Data Availability

All data supporting the findings of this study are available within the paper.

## Footnotes

1 GraphPad Prism, version 8.0; GraphPad Software Inc, La Jolla, CA.

## References

[1] Organization WH. Obesity and overweight. Fact Sheet 2021; https://www.who.int/news-room/fact-sheets/detail/obesity-and-overweight

[2] Federation WO. World Obesity Atlas. 2022.

[3] Health effects of overweight and obesity in 195 countries over 25 years. N Engl J Med. 2017;377(1):13–27. 10.1056/NEJMoa1614362 PMC5477817 28604169

[4] Chandler M (2017). Obesity and Associated Comorbidities in people and companion animals: a One Health Perspective. J Comp Pathol.

[5] Day MJ (2017). One Health Approach to preventing obesity in people and their pets. J Comp Pathol.

[6] Wall M, Cave NJ, Vallee E. Owner and cat-related risk factors for feline overweight or obesity. Front Veterinary Sci. 2019;6. 10.3389/fvets.2019.00266 PMC6709657 31482097

[7] Clark M, Hoenig M (2021). Feline comorbidities: pathophysiology and management of the obese diabetic cat. J Feline Med Surg.

[8] Banfield. New data reveals pet obesity epidemic existed long before quarantine 2021 accessed August 3, 2022]; https://www.banfield.com/en/about-banfield/newsroom/press-releases/2021/new-data-reveals-pet-obesity-epidemic-existed-long-before-quarantine

[9] Banfield. Obesity in dogs and cats, in Obesity in dogs and cats: state of pet health report. cited 24 November 2017: https://www.banfield.com/state-ofpet-health/obesity

[10] Dowgray N (2022). Aging in cats: owner observations and clinical finding in 206 mature cats at enrolment to the cat prospective aging and Welfare Study. Front Vet Sci.

[11] Kopelman PG (2000). Obesity as a medical problem. Nature.

[12] Zoran DL (2010). Obesity in dogs and cats: a metabolic and endocrine disorder. Vet Clin North Am Small Anim Pract.

[13] Clark M, Hoenig M (2016). Metabolic effects of obesity and its Interaction with Endocrine diseases. Vet Clin North Am Small Anim Pract.

[14] Global BMIMC (2016). Body-mass index and all-cause mortality: individual-participant-data meta-analysis of 239 prospective studies in four continents. Lancet.

[15] Schwartz MW (2017). Obesity pathogenesis: an endocrine Society Scientific Statement. Endocr Rev.

[16] Crewe C, An YA, Scherer PE (2017). The ominous triad of adipose tissue dysfunction: inflammation, fibrosis, and impaired angiogenesis. J Clin Invest.

[17] Ryan DH, Yockey SR (2017). Weight loss and improvement in Comorbidity: differences at 5%, 10%, 15%, and over. Curr Obes Rep.

[18] Powell-Wiley TM (2021). Obesity and Cardiovascular Disease: A Scientific Statement from the American Heart Association. Circulation.

[19] Muller TD (2022). Anti-obesity drug discovery: advances and challenges. Nat Rev Drug Discov.

[20] Larsen JA (2017). Risk of obesity in the neutered cat. J Feline Med Surg.

[21] Loftus JP, Wakshlag JJ (2015). Canine and feline obesity: a review of pathophysiology, epidemiology, and clinical management. Vet Med (Auckl).

[22] Muller TD (2019). Glucagon-like peptide 1 (GLP-1). Mol Metab.

[23] Gilor C, Rudinsky AJ, Hall MJ (2016). New approaches to Feline Diabetes Mellitus: glucagon-like peptide-1 analogs. J Feline Med Surg.

[24] Su N (2016). Exenatide in obese or overweight patients without diabetes: a systematic review and meta-analyses of randomized controlled trials. Int J Cardiol.

[25] Domecq JP (2015). Clinical review: drugs commonly associated with weight change: a systematic review and meta-analysis. J Clin Endocrinol Metab.

[26] *Victoza [package insert]. Plainsboro, NJ: Novo Nordisk Inc. 2019*

[27] Wilding JPH, et al. Once-weekly semaglutide in adults with overweight or obesity. New England Journal of Medicine; 2021. 10.1056/NEJMc2106918 34192450

[28] Blundell J (2017). Effects of once-weekly semaglutide on appetite, energy intake, control of eating, food preference and body weight in subjects with obesity. Diabetes Obes Metab.

[29] Verdich C (2001). The role of postprandial releases of insulin and incretin hormones in meal-induced satiety–effect of obesity and weight reduction. Int J Obes Relat Metab Disord.

[30] Gilor C (2011). The GLP-1 mimetic exenatide potentiates insulin secretion in healthy cats. Domest Anim Endocrinol.

[31] Scuderi MA (2018). Safety and efficacy assessment of a GLP-1 mimetic: insulin glargine combination for treatment of feline diabetes mellitus. Domest Anim Endocrinol.

[32] Kramer AL (2020). Glycemic variability in newly diagnosed diabetic cats treated with the glucagon-like peptide-1 analogue exenatide extended release. J Vet Intern Med.

[33] Klotsman M et al. Safety, tolerability, and proof-of-concept study of okv-119, a novel exenatide long-term drug delivery system, in healthy cats. Front Veterinary Sci. 2021;8. 10.3389/fvets.2021.661546 PMC8144329 34046446

[34] Schneider EL (2019). A once-monthly GLP-1 receptor agonist for treatment of diabetic cats. Domest Anim Endocrinol.

[35] Lakens D (2013). Calculating and reporting effect sizes to facilitate cumulative science: a practical primer for t-tests and ANOVAs. Front Psychol.

[36] Heymsfield SB, Wadden TA (2017). Mechanisms, pathophysiology, and management of obesity. N Engl J Med.

[37] Smith GI, Mittendorfer B, Klein S (2019). Metabolically healthy obesity: facts and fantasies. J Clin Invest.

[38] Grunvald E (2022). AGA Clinical Practice Guideline on pharmacological interventions for adults with obesity. Gastroenterology.

[39] Arnett DK (2019). 2019 ACC/AHA Guideline on the primary Prevention of Cardiovascular Disease: executive summary: a report of the American College of Cardiology/American Heart Association Task Force on Clinical Practice guidelines. Circulation.

[40] Buse JB (2010). DURATION-1: Exenatide once Weekly produces sustained glycemic control and weight loss over 52 weeks. Diabetes Care.

[41] Dushay J (2012). Short-term exenatide treatment leads to significant weight loss in a subset of obese women without diabetes. Diabetes Care.

[42] Astrup A (2009). Effects of liraglutide in the treatment of obesity: a randomised, double-blind, placebo-controlled study. Lancet.

[43] Pi-Sunyer X (2015). A Randomized, Controlled Trial of 3.0 mg of Liraglutide in Weight Management. N Engl J Med.

[44] Davies MJ (2015). Efficacy of Liraglutide for Weight loss among patients with type 2 diabetes: the SCALE Diabetes Randomized Clinical Trial. JAMA.

[45] Van de Velde H (2013). The cat as a model for human obesity: insights into depot-specific inflammation associated with feline obesity. Br J Nutr.

[46] Okada Y (2017). Comparison of visceral Fat Accumulation and Metabolome markers among cats of varying BCS and novel classification of feline obesity and metabolic syndrome. Front Vet Sci.

[47] Okada Y (2019). Diagnostic criteria for obesity disease in cats. Front Vet Sci.

[48] Williams MC (2019). Association of circulating adipokine concentrations with indices of adiposity and sex in healthy, adult client owned cats. BMC Vet Res.

[49] Wing RR (2011). Benefits of modest weight loss in improving cardiovascular risk factors in overweight and obese individuals with type 2 diabetes. Diabetes Care.

[50] Rosenstock J (2018). Efficacy and safety of ITCA 650, a Novel drug-device GLP-1 receptor agonist, in type 2 diabetes uncontrolled with oral antidiabetes drugs: the FREEDOM-1 trial. Diabetes Care.

[51] Henry RR (2014). Continuous subcutaneous delivery of exenatide via ITCA 650 leads to sustained glycemic control and weight loss for 48 weeks in metformin-treated subjects with type 2 diabetes. J Diabetes Complicat.

[52] Aroda VR (2018). A review of GLP-1 receptor agonists: evolution and advancement, through the lens of randomised controlled trials. Diabetes Obes Metab.

