# Supplementary material for: Drug release profile of a novel exenatide long-term drug delivery system (OKV-119) administered to cats
Source: BMC Vet Res. 2024 May 18;20:211. doi: 10.1186/s12917-024-04051-6 (PMC11102179; doi:10.1186/s12917-024-04051-6)

Supplemental Figure 1: Implanted OKV-119 systems in five purpose-bred cats. Images were taken on Day 112 prior to the removal procedure.

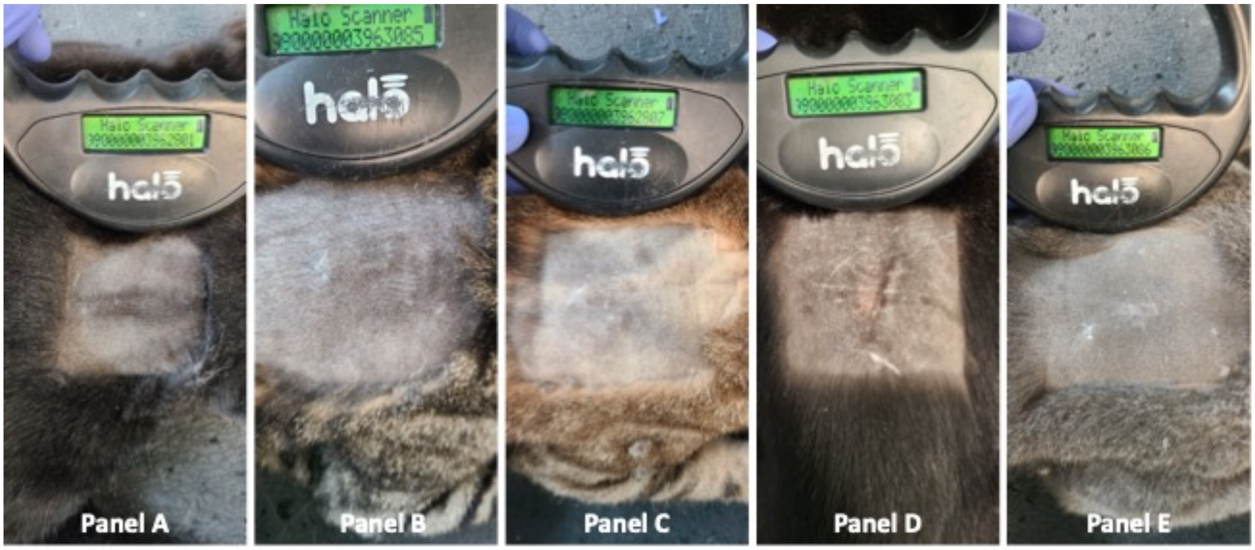

Supplement: Supplementary file 1 — Supplementary Material 1 [file 12917_2024_4051_MOESM1_ESM.pdf]
